# Chinese economic development difference factors empirical study

**DOI:** 10.1371/journal.pone.0319957

**Published:** 2025-05-23

**Authors:** Lihua Ma, Mingmei Sun, Huizhe Yan, Yufei Chen

**Affiliations:** 1 School of Management Engineering and Business, Hebei University of Engineering, Handan, China; 2 Uibe Business School, University of International Business and Economics, Beijing, China; Thammasat University, THAILAND

## Abstract

This article provides a detailed analysis and empirical support for the fiscal decentralization system, exploring its mechanisms in regulating the imbalance and insufficiency of regional economic development in China. The research found that fiscal decentralization, as a social governance tool, has a significant positive effect on narrowing the economic development gap between cities and addressing developmental disparities, particularly in the southern and vast inland regions of China. This effect provides important impetus for regional economic balance and comprehensive development. Secondly, mechanism testing revealed that technological support and the optimization of the administrative environment play crucial roles in promoting balanced regional economic development. By optimizing the industrial structure and strengthening the regulatory strategy for fiscal pressure, the positive impact of fiscal decentralization on economic development can be significantly enhanced. Furthermore, this study employs panel data threshold effect analysis to reveal that the level of tax competition moderates the effects of fiscal decentralization within a certain range, which in turn impacts the trajectory of economic development. These insights aim not only to leverage the fiscal decentralization system to alleviate and improve the uneven and inadequate economic development among regions in China and help achieve the goal of social governance, but also to provide a solid theoretical basis and practical guidance for the government in designing more precise and effective economic policies, which holds significant strategic importance and practical value.

## 1 Introduction

In the context of global economic integration and regional coordinated development, uneven and insufficient economic development has always been one of the core issues of concern for governments and scholars around the world. As the world's largest developing economy, China also faces a series of challenges. Fiscal decentralization plays a key role in promoting balanced economic development and achieving comprehensive prosperity. In the face of imbalances and inadequacies in economic development, in-depth research on the relationship between fiscal decentralization and economic development has important theoretical significance and practical guidance value for building a more fair and equitable fiscal system and promoting the realization of common prosperity and improving the level of social governance for all citizens.

Fiscal decentralization is an important institutional arrangement that achieves balanced and sufficient economic development by delegating fiscal power to local governments. Its impact on economic development is a complex and important issue. On the one hand, fiscal decentralization endows local governments with more financial resources and power, enabling them to better meet the natural and cultural needs of the region [1] and promote economic growth [2–4]. On the other hand, fiscal decentralization also poses some potential problems and challenges. When facing financial pressure, local governments may have a tendency to excessively pursue economic growth, neglecting environmental protection and social equity, which can lead to imbalanced and insufficient economic development.

While the effects of fiscal decentralization on economic development have been extensively studied and discussed, it is worth noting that most scholars have focused more on the impact of fiscal decentralization on overall economic performance and income inequality when exploring this issue, while few have explicitly studied its effect on uneven and insufficient economic development. Some scholars believe that fiscal decentralization is beneficial for economic development. Pommerehne [5] and Panizza [6] argue that there is a positive correlation between a country's level of economic development and the degree of decentralization in the public sector. Qian and Weingast’s [7] study first explored the complex relationship between fiscal decentralization and economic growth, founding that fiscal decentralization can be a powerful factor driving long-term economic prosperity. Subsequent research by Akai and Sakata [8] supports this viewpoint, emphasizing the positive role of fiscal decentralization in promoting sustained economic growth. Another opposing view suggests that delegating fiscal power may hinder economic development. Arung Lamba et al. [9] conducted regression analysis to demonstrate that fiscal decentralization policies have affected economic growth in eastern Indonesia, while confirming that these policies have not alleviated economic imbalances in the region. Hung and Thanh [10] investigated the combined effects of fiscal decentralization on economic growth and human development, concluding that it positively influences economic growth. Additionally, other researchers have explored how financial decentralization affects income inequality. Some scholars content that local governments have dispersed their expenditures, leading to economic disparities between different regions [11–12]. However, others have also proposed that through empirical analysis of economic differences among regions, varying conclusions have been drawn, namely that there are different degrees of economic disparities between regions [13].

Song [12] explores how fiscal decentralization contributes to rising regional income inequality in China, while Liu et al. [14] analyze the interplay between fiscal decentralization, equalization, and intra-provincial inequality, offering valuable insights into their effects on economic development. But there are still some areas that have not been fully explored and unresolved issues. This article seeks to explore how China’s fiscal decentralization affects uneven and inadequate economic development, as well as its role and mechanism in promoting common prosperity, through theoretical exploration and empirical analysis. Explored how fiscal decentralization affects this imbalance and systematically explained its regulatory role on industrial structure and fiscal pressure. To achieve this goal, we have employed various statistical tools and econometric models, including panel data regression analysis, threshold effect models, and instrumental variable methods. These tools have been carefully selected to address potential endogeneity issues, control regional heterogeneity, and ensure the robustness of our research results. By utilizing these advanced statistical techniques, our goal is to provide a more detailed understanding of the complex relationship between fiscal decentralization and economic development, thereby providing more precise and actionable policy recommendations.

The research contribution of this article lies in: (1) Exploring new perspectives. This article focuses on the impact of fiscal decentralization on uneven and insufficient economic development, which has been less systematically explored in previous literature. Most studies focus on the impact of fiscal decentralization on overall economic performance or income inequality, while this article fills the research gap on its effects on uneven and insufficient economic development, providing a new theoretical perspective for understanding the economic effects of fiscal decentralization. (2) Reveal the mechanism of action and regulatory factors. This article reveals the moderating effect of tax competition level on fiscal decentralization within a moderate range, which in turn affects the trajectory of economic development. This discovery provides a new perspective and theoretical support for our understanding of how fiscal decentralization affects economic imbalances and inadequacies. (3) Innovation in variable selection. Unlike previous studies that focused on a single or few variables, this article introduces a series of new variables, such as local government size, industrial structure, and technological support, to comprehensively examine the multidimensional impact of fiscal decentralization on regional economic development. The addition of these new variables makes the analysis in this article more in-depth and comprehensive, revealing more potential impact mechanisms and pathways. (4) Expand the boundaries of research on fiscal decentralization. Through the analysis of tax competition, this article explores the complex impact of fiscal decentralization on economic development, promotes in-depth research on fiscal decentralization, and expands the boundaries of relevant theories. (5) This article has also made breakthroughs in research methods. This article adopts advanced econometric models and ensures the reliability and effectiveness of research results through rigorous empirical analysis and data validation. Meanwhile, this article also pays special attention to inter provincial correlation issues and adjusts the regression model through clustering standard error method to ensure the robustness of the research conclusions. (6) Endogenous issues. Previous studies have had endogeneity issues. We addressed this potential endogeneity problem by using instrumental variable estimation in regression analysis, using the average fiscal decentralization power of provinces outside the studied province as a tool to measure fiscal decentralization in that province.

## 2 Literature review

Fiscal decentralization, as a global political and economic trend, has become an important tool for governments to regulate economic operations and optimize resource allocation [15]. It not only clarifies the fiscal relationships between different levels of government [16,17], but also plays an irreplaceable role in promoting the coordination of economic functions among governments at all levels and leveraging their respective comparative advantages. The decentralization of fiscal power has given local governments greater autonomy and flexibility, enabling them to formulate more targeted policies based on local conditions and utilize information advantages more effectively [18]. This transformation not only improves the efficiency of the public sector [10], but also promotes the personalization and refinement of public services, better meeting the needs and preferences of the people.

The level of fiscal decentralization in developing countries is generally lower than that in developed countries [19], while China’s fiscal decentralization is significantly higher than that of other developing countries [20], largely due to differences in economic and political will, expenditure distribution, and tax systems. By increasing fiscal decentralization, developing countries can better adapt to local economic development needs, achieve balanced economic development [21], and reduce regional economic inequality [22]. A large number of empirical studies have concluded that there is a significant positive correlation between fiscal decentralization and economic growth [23][24–26]. On the one hand, local governments formulate flexible economic policies based on the characteristics and needs of the local area, promote the optimization and upgrading of the industrial structure, and enhance the competitiveness and innovation capacity of the economy. On the other hand, fiscal decentralization promotes coordination and cooperation between regions. By strengthening communication and coordination among local governments, various regions can jointly formulate strategies for cooperative development, promote the rational allocation of resources [27], and achieve balanced development among regions.

However, it's important to recognize that the effects of fiscal decentralization on the economy are not singular and absolutely positive. Liu, Martinez-Vazquez, and Wu’s [28] study used county-level data from 1995 to 2009 to analyze the impact of fiscal decentralization and equalization below the provincial level in China on intra provincial inequality. Through fixed effects models and instrumental variable methods, research found that fiscal decentralization exacerbates inequality within provinces, while the equalization policies of provincial governments help alleviate this phenomenon. Cavusoglu and Dincer [29] studied the relationship between fiscal decentralization and income inequality by using state-level data in the United States. They used the FMOLS method to analyze various inequality indicators, including the highest income share and the Theil index, and considered control variables such as education level, wage ratio, and unionization rate. The Research revealed that fiscal decentralization is effective in reducing income inequality in wealthy states but may exacerbate inequality in poor states. In addition, Scott [30] revealed through his research on the relationship between decentralization and various variables such as service provision, economic development, and social cohesion that the lack of clear statistics significantly affects the impact of decentralization on economic development. Labutková et al. [31] used two variable indicators to measure economic inequality and found through cluster analysis that countries with higher levels of decentralization belong to a group of countries that are more favorable for economic development. Siburian’s [32] study suggests a negative correlation between fiscal decentralization and regional income inequality in Indonesia. By applying the SEM combined with the GMM HAC method, it was found that decentralization significantly reduces the economic development differences between regions, while regional inequality has no significant impact on fiscal decentralization. This indicates that local governments can allocate resources more effectively based on local characteristics and reduce economic inequality. Hanif et al.[33] used a two-step system generalized method of moment estimation to analyze panel data from 15 developing countries between 2000 and 2015. The research findings indicate that fiscal decentralization significantly boosts economic growth in these federal countries. However, this study also found that the positive impact of fiscal decentralization weakens when the political environment is unstable. Jin and Rider [34] used panel data from China and India from 1985 to 2005 to examine the effects of expenditure decentralization and fiscal equalization on short-term and long-term economic growth. The study found that in India, expenditure decentralization had a positive impact on long-term economic growth, while in China, this was not the case. In the past 20 years in China, fiscal decentralization has made significant contributions to economic growth, driving economic growth [35], and promoting balanced economic development. Therefore, due to the controversy surrounding the above results and the dynamic changes in global socio-economic trends, it is essential to thoroughly examine how fiscal decentralization influences the uneven and inadequate development of the Chinese economy. The comparison with other studies is shown in Table 1.

**Table 1:** Other studies.

Author	Research variables	Research method	Conclusion	Time
Song, Y [12]	Decentralization of expenditure, decentralization of income, and autonomy of local governments	Econometric model	China’s fiscal expenditure decentralization has significantly increased income inequality between regions, and the impact of income decentralization on inequality differs before and after the tax reform in 1994. In addition, the autonomy of local governments increased regional inequality before the reform, but has a positive effect on reducing inequality after the reform.	2013
Yongzheng Liu、Jorge Martinez-Vazquez and Alfred M. Wu [14]	Fiscal decentralization, equalization, intra provincial inequality, logarithm of per capita GDP, output share of state-owned enterprises, urbanization rate, openness, and education years	Instrumental variable method	Fiscal decentralization significantly reduces income disparities between regions, while the impact of regional inequality on fiscal decentralization is not significant.	2019
Digdowiseiso、Kumba、Syed M.Murshed and Sylvia I.Bergh [36]	Fiscal decentralization, inequality, institutional quality, military expenditure, natural logarithm of per capita GDP, population growth rate, trade openness, government expenditure as a percentage of GDP	GMM	Under good institutional quality and moderate military spending, fiscal decentralization can effectively reduce income and ethnic inequality.	2022
Matondang Elsa Siburian [32]	Fiscal decentralization, Income inequality	SEM GMM HAC	Fiscal decentralization significantly reduces income disparities between regions, while the impact of regional inequality on fiscal decentralization is not significant.	2019
Nguyen, Hung Thanh, et al. [37]	Inequality within the province, fiscal decentralization, equalization efforts, per capita GDP, and the proportion of the secondary industry to GDP.	Bidirectional fixed effects model; Instrumental variable method	Fiscal decentralization in China has led to greater intra provincial inequality, which is consistent with the previous assumption that fiscal decentralization may increase regional inequality.	2020
Cavusoglu, Tarkan, and Oguzhan Dincer [29]	Fiscal decentralization, income inequality, years of education, agricultural to non-agricultural wage ratio, unionization rate, per capita GDP.	Fully Modified Ordinary Least Squares; Granger Non Causal Test	Fiscal decentralization can reduce income inequality in wealthy states, but may backfire in poorer states.	2015

Based on the above review, most research mainly focuses on the impact of fiscal decentralization on overall economic performance, lacking a systematic analysis of its role in addressing regional economic imbalances and inadequacies. Secondly, the mechanism and regulatory factors of fiscal decentralization have not been thoroughly explored. In addition, existing literature lacks sufficient analysis of the differences in the effects of fiscal decentralization in different regions and stages of economic development, resulting in limited guidance for policy-making. Based on the above limitations, this study proposes a more comprehensive theoretical framework, focusing on analyzing how fiscal decentralization can promote regional economic balance and full development through mechanisms such as technological support, administrative environment optimization, and industrial structure adjustment. On the theoretical basis, the specific mechanism by which fiscal decentralization affects the uneven and insufficient economic development in China’s regions was thoroughly analyzed. Through detailed theoretical analysis and empirical research, this study not only verifies the key role of fiscal decentralization in alleviating regional economic development gaps and deficiencies, but also reveals the regulatory function of tax competition level on the effectiveness of fiscal decentralization within a moderate range, which in turn affects the trajectory of economic development. Meanwhile, new research variables have been added. Therefore, this study not only fills the gap in previous research in terms of theoretical contribution, but also provides important references and inspirations for the optimization of fiscal decentralization system and the balanced development of regional economy.

## 3 Heoretical analysis and research hypothesis

Fiscal decentralization, as an institutional arrangement for intergovernmental fiscal relations, lies at the core of transferring some fiscal powers and responsibilities from the central authority to local administrations [10]. It aims to enhance policy implementation efficiency, promote optimal resource distribution, and stimulate regional economic development [38]. However, the relationship between fiscal decentralization and economic growth is not linear; it is regulated and influenced by multiple factors, among which the government environment, as one of the key factors, cannot be ignored. From a theoretical perspective, the government environment encompasses multiple dimensions such as governance efficiency, policy implementation capacity, institutional transparency, and stability. These factors directly affect whether fiscal decentralization policies can be effectively implemented and achieve expected results. An efficient government environment can reduce information asymmetry and moral hazard in the process of policy implementation, enhance the policy response and innovation capabilities of local governments, and thus amplify the positive effect of fiscal decentralization on economic growth. Looking back at existing literature, it is not difficult to find that there is significant spatial heterogeneity in the impact of fiscal decentralization on economic growth. On the one hand, fiscal decentralization empowers local governments with more fiscal autonomy, incentivizing them to formulate more flexible and effective economic policies based on local conditions, and promoting local economic growth [39,40]; On the other hand, while pursuing economic growth, local governments may also adopt short-sighted decisions due to performance evaluation pressure, such as excessive competition for tax sources and neglecting long-term sustainable development, leading to resource misallocation and regional development imbalance. At this point, the quality of the government environment becomes the key to regulating this contradiction.

The government environment also indirectly affects regional economic growth by influencing the environmental governance behavior of local governments. Under the fiscal decentralization system, local governments often face trade-offs between environmental protection and economic development. A positive government environment can encourage local governments to pay more attention to environmental protection and sustainable development while pursuing economic growth. By increasing investment in environmental protection and enhancing environmental governance capabilities, green economic growth can be achieved. On the contrary, if the government environment is poor, local governments may neglect environmental protection in favor of by short-term interests, leading to environmental degradation and unsustainable economic growth. The importance of the government environment as a moderating factor between fiscal decentralization and economic growth is self-evident. In empirical research, by developing an analytical framework that incorporates government environmental factors, the intricate impact mechanism of fiscal decentralization on China’s regional economic development can be more thoroughly understood, offering a stronger scientific basis for policymakers’ decisions. Therefore, hypothesis H1 is proposed:

H1: The government environment was the moderation factor between fiscal decentralization and economic growth

Fiscal decentralization, as an important component of the modern national governance system, lies at the core of empowering local governments with more fiscal power and responsibility, stimulating their enthusiasm and creativity, and promoting local economic development in a more flexible and efficient way [34]. In the study of the impact of fiscal decentralization on regional economic development in China, fiscal decentralization plays an important role in reducing imbalances and deficiencies in economic development. Firstly, from the perspective of resource allocation efficiency, fiscal decentralization helps optimize resource allocation and reduce resource waste. Under a highly centralized fiscal system, the central government may find it difficult to fully understand the actual situation and needs of various regions, resulting in uneven and inefficient resource allocation. Fiscal decentralization, by granting local governments greater fiscal autonomy, enables them to allocate resources based on local conditions, improve the targeted and effective use of resources, and thus help reduce imbalances in economic development [36]. Secondly, fiscal decentralization can motivate local governments to actively participate in economic development and promote balanced regional economic growth [41]. Under the fiscal decentralization system, local governments actively seek opportunities for economic growth, increase investment in infrastructure, education, healthcare, and other fields, improve the investment environment, attract external capital and technology, and promote the sustained and healthy development of the local economy in order to obtain more fiscal revenue and better performance. This active participation behavior helps narrow the development gap between regions and reduce deficiencies in economic development. However, fiscal decentralization may also bring some potential problems. Under fiscal decentralization, local governments may compete for limited resources and funds for their own interests, especially when the overall government budget is limited. This competition may exacerbate regional inequality and funding shortages. The allocation of funds is often influenced by government priorities and political considerations, which may result in certain regions receiving more resources while others face funding shortages. Therefore, while fiscal decentralization promotes economic development, it may also exacerbate regional inequality and uneven resource allocation. Furthermore, fiscal decentralization can promote competition and cooperation among local governments, forming a development pattern of positive interaction [42,43]. Under the fiscal decentralization system, local governments engage in fierce competition for limited resources and markets. This competition helps to stimulate the innovative spirit and reform drive of local governments, promoting their continuous optimization of the policy environment and service level. At the same time, local governments will also work together to solve cross-regional economic development problems through cooperation and communication, achieve resource sharing and complementary advantages, and further reduce imbalances and deficiencies in economic development. Therefore, hypothesis H2 is proposed:

H2:Fiscal decentralization can effectively reduce imbalances and deficiencies in economic development.

## 4 Research design

### 4.1 Model setting.

Digdowiseiso et al.[36] used the GMM approach to analyze the effects of fiscal decentralization on inequality in developing countries; Thanh [37] used using 3SLS-GMM and GMM-HAC models to study the connection between fiscal decentralization and income inequality in different Vietnamese provinces. This article focuses on the influence of financial decentralization on the unbalanced and inadequate development of economy. Base on the research purpose of this paper, a benchmark model was constructed, as shown in equation (1):


$$Eco_{it}=\alpha_{0}+\alpha_{1}FA_{it}+\theta Xit+\eta_{i}+\lambda_{t}+\varepsilon_{it}$$ (1)

Here, $\alpha_{0}$ is the constant term, where Eco measures an indicator of uneven and insufficient economic development, FA represents fiscal decentralization, X represents provincial fixed effects, η represents a fixed year, and ε is an error term.


$$M_{it}=\alpha_{0}+\alpha_{1}AE_{it}+\theta Xit+\eta_{i}+\lambda_{t}+\varepsilon_{it}$$ (2)

$$Eco_{it}=\alpha_{0}+\alpha_{1}FA_{it}+\alpha_{2}M_{it}+\theta Xit+\eta_{i}+\lambda_{t}+\varepsilon_{it}$$ (3)

Fiscal decentralization effect onto Chinese economy uneven and imbalanced development role could be described by combing Equations (1)–(3). Additionally, this project will create model (4) to empirically test the regulatory effects of factors like industrial structure and fiscal pressure on intergovernmental fiscal decentralization.


$$Eco_{it}=\alpha_{0}+\alpha_{1}FA_{it}+\alpha_{2}MV_{it}+\alpha_{3}FA_{it}\times MV_{it}+\theta Xit+\eta_{i}+\lambda_{t}+\varepsilon_{it}$$ (4)

On this basis, this article constructs a “dual threshold effect” model to verify the “threshold effect” of tax competition in relation to fiscal decentralization. Tax competition is used as a threshold variable, withω_*1*_ and ω_*2*_ set as threshold values.


$$\begin{array}{c} Eco_{it}=\alpha_{0}+\alpha_{1}FA_{it}\cdot I(CB_{it}\leq \omega_{1})+\alpha_{2}FA_{it}\cdot I(\omega_{1}<CB_{it}\leq \omega_{2})\\ +\alpha_{3}FA_{it}\cdot I(CB_{it}>\omega_{2})+\theta Xit+\eta_{i}+\lambda_{t}+\varepsilon_{it} \end{array}$$ (5)

In model Equation (1), Eco represents an indicator of economic imbalance and insufficiency, FA represents fiscal decentralization, X represents control variables used to control for other factors that may affect economic imbalance and insufficiency, and ε it is the error term. Specifically, Ecoit can be further decomposed into two sub indicators: Economic Imbalance (EI) and Economic Shortage (EH). Economic imbalance (EI) is usually measured by differences in economic growth between cities, while economic insufficiency (EH) is measured by indicators such as scope standardization (EH_S). In model Equations (2) and (3), Mit represents mechanism variables such as technical support (TS) and administrative environment (CF), which mediate the impact of fiscal decentralization on economic imbalances and deficiencies. FAit represents fiscal decentralization, MVit represents moderating variables such as industrial structure (IB) and fiscal pressure (PS), which regulate economic imbalances and deficiencies by influencing the effect of fiscal decentralization. In model Equation (4), the moderating effect was further introduced to examine the moderating effects of industrial structure (IB) and fiscal pressure (PS) on fiscal decentralization. Specifically, the Industrial Structure (IB) enhances the alleviating effect of fiscal decentralization on economic imbalances and deficiencies by optimizing resource allocation and promoting industrial upgrading. Fiscal pressure (PS) regulates the impact of fiscal decentralization on economic development by influencing the fiscal behavior of local governments. In model Equation (5), tax competition (CB) was introduced as a threshold variable to examine the impact of fiscal decentralization on economic imbalances and deficiencies at different levels of tax competition. Tax competition (CB) regulates the effect of fiscal decentralization on economic development by influencing the competitive behavior among local governments. When tax competition is at a low level, fiscal decentralization has a weaker mitigating effect on economic imbalances and deficiencies; When tax competition is at a high level, the effect of fiscal decentralization is significantly enhanced.

### 4.2 Variable setting.

The two main explanatory factors are uneven and inadequate economic growth. To evaluate the imbalance, benchmark analysis relies on the differences in economic growth between cities within a province, represented as EI’a. To ensure the robustness of our research results, robustness tests included other indicators, namely Gini coefficient (EI-G) and Theil index (EI_T), which provide different perspectives on economic inequality. Regarding the issue of insufficient economic development, benchmarking uses scope standardization (EH_S) as the main measure to capture the degree to which the economy has not reached its potential. To further validate our analysis, robustness testing introduced linear proportional change (EH_L) and benchmark transformation (EH_S) as complementary indicators, each providing a unique perspective to evaluate economic deficiencies. The symbols in the formulas are defined as follows: Y represents the actual per capita Gross Domestic Product (GDP) of the province, indicating economic performance per person, while N denotes the province's total population at year-end, which is used to calculate per capita metrics, ${\overset{―}{Y}}_{it}=\sum_{j=1}^{ci}n_{jt}\;\;╱\;\;N_{it}\;\times y_{jt}$ represents the per capita GDP of the province based on the urban population proportion, while L signifies the number of employed individuals. The subscripts used in the formulas have distinct meanings: ‘t’ designates the specific year being referenced, ‘j’ and ‘k’ represent individual cities within a broader context, ‘i’ signifies a particular province, ‘max’ and ‘min’ indicate the upper and lower bounds of economic development levels, with ‘base period’ referring to a fixed reference point for comparison, and ‘s’ designates different industries or sectors.

Fiscal decentralization, as a pivotal explanatory variable, encompasses diverse quantification approaches such as income, expenditure, and autonomy indices. Our findings emphasize the pronounced influence of both the “autonomy index” and “income index” on model validations, with the “fiscal autonomy” index standing out as an effective barometer for regional disparities. However, it is acknowledged that the fiscal autonomy metric has inherent constraints in capturing temporal shifts in decentralization dynamics. To mitigate this limitation and ensure robustness, this study adopts the “Autonomy Index (AI)” as the primary benchmark for regression analysis. Additionally, we introduce “Fiscal Revenue Diversification (DF)” and “Fiscal Relative Diversification (DB)” as complementary measures for validation. The concept of “DB” draws inspiration from CDB et al.‘s methodology for assessing fiscal vertical imbalance, offering a relative perspective on the dispersion patterns of fiscal revenues and expenditures, thereby enhancing our ability to comprehensively evaluate fiscal decentralization trends.

Apart from the dependent and explanatory variables, a comprehensive array of mechanism, moderating, and threshold variables have been incorporated, all of which potentially influence the phenomenon of imbalanced and inadequate economic development, including financial development (NF), urbanization (CU), industrial development (DS), macro tax burden (MTB), inflation (IH), government size (GS), non tax work (NTW), opening up to the outside world (OW), and technology support (TS) Administrative environment (CF), industrial structure (IB), financial pressure (PS), and tax competition (CB).

In order to preliminarily explore the relationship between the main variables, we calculated the Pearson correlation coefficients between these variables and constructed a correlation matrix (Table 2). The results showed a significant positive correlation between fiscal decentralization and underdevelopment (r  =  0.23, p  <  0.01), indicating a strong linear relationship between the two. At the same time, we also noticed a high correlation between economic imbalances and variable financial development (r  =  0.75, p  <  0.01), which may suggest the existence of multicollinearity and needs to be noted in subsequent analysis.

**Table 2:** Pearson correlation coefficient matrix of the main variables.

Variable	Fiscal decentralization	Insufficient economic development	Economic imbalances	Financial development	Urbanization	Industrial structure
Fiscal Decentralization	1.00	0.23*	0.35	0.33	0.44*	0.31*
Insufficient economic development	0.53*	1.00	0.55	0.11*	0.67	0.32
Economic imbalances	0.44*	0.43	1.00	0.75*	0.56*	0.39*
Financial Development	0.56	0.21*	0.34*	1.00	0.33	0.33
Urbanization	0.50*	0.23*	0.54	0.44*	1.00	0.42
Industrial structure	0.74	0.42	0.63	0.55*	0.41*	1.00

The closer the correlation coefficient is to 1 or -1, the stronger the correlation between the two coefficients, and the closer it is to 0, the weaker the correlation. The coefficient between economic imbalance and financial development is 0.75, indicating multicollinearity between the two variables. Multicollinearity can cause variance inflation in ordinary least squares estimation, reducing the stability and explanatory power of regression coefficients. Ridge regression reduces variance by introducing a bias term (K-value), providing more stable estimation results in the presence of multicollinearity. The criterion for selecting the K value is to significantly reduce the variance of the regression coefficients while avoiding introducing excessive bias. The independent variables are insufficient economic development, economic imbalance, financial development, urbanization, and industrial structure, and fiscal decentralization is the dependent variable. When the K value increases to 0.1, the regression coefficients of each variable tend to stabilize, indicating that the balance between bias and variance is optimal at this time. The results of ridge regression are shown in Table 3. The R2 of the regression model is 0.994, indicating a good fit and significant influence of all variables.

**Table 3:** Ridge regression results.

	R		R^2^	Adjust R^2^	
	0.995		0.994	0.983	
Variable	Non standardized coefficient		Standardization coefficient	t	p
	B	standard error	Beta		
constant		2.076		3.238	0.018
Insufficient economic development	0.352	0.030	0.269	4.902	0.001
Economic imbalances	0.342	0.052	0.170	3.620	0.012
Financial Development	0.225	0.143	0.088	2.239	0.066
Urbanization	0.502	0.364	0.084	2.853	0.029
Industrial structure	0.483	0.291	0.037	2.936	0.014

### 4.3 Data sources and descriptive statistics.

This article utilizes data from three levels: provincial, municipal, and national. City-level statistical data is primarily sourced from the CEIC database. Additionally, national, provincial, and municipal data come from various sources, including the China Statistical Yearbook, China Financial Yearbook, the official website of the National Bureau of Statistics, and the EPS database. At present, the economic differences between provinces and cities in China are mainly based on the city level. In this study, relevant data from the four municipalities directly under the central government were excluded due to differences in administrative divisions compared to various provinces and cities nationwide. Finally, empirical research will be conducted on 27 provinces and cities in China. This study selected 736 observational data from 2013 to 2022.

Table 4 shows the descriptive statistics of these variables. These variables include economic imbalances, underdevelopment, fiscal decentralization, NF, CU, DS, MTB, IH, IZE, NTW, OW, TS, CF, IB, PS, and CB. Each variable has 736 observations. From the perspective of average values, economic imbalances include economic development gap (EI-A), Gini coefficient (EI_G), and Theil index (EI-T), with average values of 0.254, 0.385, and 0.284, respectively; Insufficient development includes range standardization (EH-R), linear proportional change (EH-L), and benchmark transformation (EH-F), with average values of 0.573, 0.883, and 2.834, respectively; Fiscal decentralization includes autonomous indicators (AI), fiscal revenue decentralization (DF), and fiscal relative decentralization (DB), with an average of 0.573, 0.254, and 0.634, respectively; NF is 0.038, DS is 0.453, MTB is 0.094, TS is 0.725, IB is 0.938, and so on. For these variables, the standard deviation ranges from 0.019 to 2.256. The minimum and maximum values provide the range of values for each variable. For example, the minimum value of EI_1 is 0.125 and the maximum value is 0.938. The minimum value of EH-R is 0, and the maximum value is 1. Through these descriptive statistical data, we can understand the distribution and characteristics of each variable, as well as their differences and ranges of change.

**Table 4:** Variable descriptive statistics.

Variables		Sample size	Mean	Standard deviation	Minimum	Maximum
Economic imbalance	EI_A	736	0.254	0.766	0.125	0.938
	EI_T	736	0.284	0.083	0.028	0.635
	EI_G	736	0.385	0.059	0.036	0.256
Inadequate development	EH_R	736	0.573	0.638	0	1
	EH_F	736	2.834	0.976	1	5.375
	EH_L	736	0.883	0.294	0.0983	1
Fiscal decentralization	AI	736	0.573	0.128	0.0731	0.938
	DB	736	0.634	0.194	0.282	0.829
	DF	736	0.254	0.073	0.294	0.573
Control variables	NTW	736	0.843	0.577	0.763	1.386
	IH	736	0.736	2.256	-7.725	5.386
	MTB	736	0.094	0.137	0.019	0.215
	DS	736	0.453	0.083	0.284	0.284
	CU	736	0.942	0.173	0.238	0.833
	OW	736	0.436	0.236	0.0225	1.225
	NF	736	0.038	0.019	0.024	0.085
	GS	736	0.294	0.294	0.0334	1.435
Mechanism variables	CF	736	3.046	1.394	-0.795	5.938
	TS	736	0.725	0.049	0.043	0.046
Regulating variables	PS	736	0.846	0.096	0.294	0.328
	IB	736	0.938	0.455	0.711	2.539
Threshold variable	CB	736	1.436	0.273	0.638	1.994

## 5 Results and discussion

### 5.1 Regression model and clustering standard error.

Table 5 presents a regression analysis of the uneven development of China’s economy and the mutual influence between the two. Empirical analysis shows that in the case of imbalanced economic development (the first item), the regression coefficient of fiscal decentralization reaches -0.438, passing the 5% significance test, indicating that fiscal decentralization plays a positive role in reducing economic development imbalances among provinces. In reducing the insufficient development of provinces (item 2), the decentralization of government power has a significant impact on mitigating the internal economic development disparities of provinces (at a 1% significance level), indicating that the decentralization of local government power has a significant impact on addressing the internal economic development of provinces. The results of the regression analysis confirmed the correctness of H2.

**Table 5:** Baseline regression results.

Variables	Economic imbalance	Inadequate development
EI_A	EH_R
AI	-0.438^**^	-0.076^***^
	(-1.76)	(-4.289)
NF	0.294	1.834^***^
	(0.94)	(3.143)
CU	-0.137	-0.038
	(-0.762)	(-1.284)
DS	0.771^***^	0.728^***^
	(1.797)	(-3.94)
MTB	0.046	2.553^*^
	(0.076)	(8.81)
IH	0.046	0.043
	(0.735)	(0.91)
GS	-0.453^***^	0.043
	(-5.37)	(0.735)
NTW	-0.046	-0.294
	(-0.942)	(-0.38)
OW	0.799^**^	0.024
	(6.756)	(0.538)
Constant term	0.299^***^	1.285^***^
	(13.72)	(23.94)
Province fixed effect	Yes	Yes
Year fixed effect	Yes	Yes
Observed values	736	736
Adjusted R^2^	0.694	0.834

Note:^***^, ^**,*^ represents the regression coefficients at significance levels of 1%, 5%, and 10%, robust T-values are shown in parentheses.

To ensure the robustness of the regression results, we further incorporated cluster adjustments into the regression model to account for potential intra-provincial correlations that might affect the standard errors. Specifically, we clustered the standard errors at the provincial level, which can effectively explain and correct standard error deviations that may arise due to similarity or policy consistency within the province. This adjustment is not only a standard practice in regional research, but also helps us obtain more accurate and reliable statistical inferences. Next, to better understand how fiscal decentralization affects economic development across different regions, we grouped 27 provinces (autonomous regions) based on their similarities in economic imbalance (EI) and insufficientdevelopment (EH). Through clustering analysis or similarity evaluation methods, we divided these provinces into four main groups, and the provinces within each group showed high similarity in terms of economic inequality and insufficient development. The results are shown in Table 6.

**Table 6:** Cluster analysis.

Group	Province List	Mean of Economic Imbalance Index (EI)	Economic Underdevelopment Index (EH) Mean
1 (Province with the highest average economic imbalance)	Yunnan, Guizhou, Fujian, Hainan, Xizang, Xinjiang	47.6	56.8
2 (Province with the highest average degree of decentralization of power)	Shanxi, Shandong, Hebei, Henan, Anhui, Jiangsu, Hubei, Liaoning	44.5	65.2
3 (Provinces with the most unequal distribution of average wealth)	Hunan, Jilin, Heilongjiang, Zhejiang, Gansu, Qinghai	65.2	44.7
4 (Province with the lowest average degree of decentralization of power)	Shaanxi, Inner Mongolia, Guangdong, Guangxi Zhuang, Sichuan, Yunnan, Ningxia Hui	58.4	43.6

### 5.2 Robustness test.

#### 5.2.1 Re-measure the indicators of imbalance and inadequacy

To ensure the reliability and consistency of the baseline regression results, we conducted robustness checks and addressed imbalanced and insufficient variables.We use the approach of Liu et al. [28], this study decomposes economic development imbalances into Gini coefficient (EI _ G) and Theil (EI _ T) and economic underdevelopment into linear proportional change (EH _ L) and base transformation (EH _ S). These new indicators give different perspectives on imbalances and gaps in economic growth. The Gini coefficient can capture the concentration of income distribution, while the Theil index is more sensitive to changes in high-income groups. The two complement the inter city difference indicators in the benchmark model, ensuring that the conclusions are not affected by a single measurement method. In the regression results of Table 7, in the indicator of economic imbalance, the regression coefficients of fiscal decentralization on EI_G and EI_T are -0.538 and -0.355, respectively, passing the significance level tests of 5% and 10%, indicating that fiscal decentralization has played a positive role in reducing economic development imbalance; In the indicator of economic inadequacy, the regression coefficients of fiscal decentralization on EH_L and EH_F are -0.732 and -0.325, respectively, and both have passed the significance level test of 5%, indicating that fiscal decentralization reduces the extent of economic development inadequacy. This is consistent with the benchmark regression results in Table 5, further confirming the positive impact of fiscal decentralization on economic development.

**Table 7:** Re-measures the regression results of imbalance and inadequacies.

Variables	Economic imbalance		Insufficient development	
EI_G	EI_T	EH_L	EH_F
AI	-0.538^**^	-0.355^*^	-0.732^**^	-0.325^**^
	(-4.07)	(-3.45)	(-5.37)	(1.27)
Control variables	Yes	Yes	Yes	Yes
Province fixed effect	Yes	Yes	Yes	Yes
Year fixed effect	Yes	Yes	Yes	Yes
Observed values	736	736	736	736
Adjusted R^2^	0.946	0.529	0.835	0.437

Note:^***^, ^**,*^ represents the regression coefficients at significance levels of 1%, 5%, and 10%, robust T-values are shown in parentheses.

The introduction of Gini coefficient and Theil index to measure the imbalance of economic development, as well as linear proportional changes and baseline transformations to measure the inadequacy of economic development, further emphasizes the importance of the fiscal decentralization system in achieving balanced economic development and sustainable growth. Fiscal decentralization provides local governments with greater autonomy and decision-making power, enabling them to better adapt to local economic characteristics and needs. By decentralizing fiscal power, local governments can more effectively promote balanced economic development in various regions and reduce imbalances and inadequacies.

#### 5.2.2 Reassess fiscal decentralization metrics

To assess the impact of fiscal decentralization on economic development, we re-evaluated the fiscal decentralization indicator by substituting alternative explanatory variables. Specifically, we replaced the original measures with two new indicators: fiscal revenue decentralization (DF) and fiscal relative decentralization (DB). The regression results of this adjustment are shown in Table 8. The regression coefficient for fiscal revenue decentralization in relation to the economic development gap is -0.284, which passed the significance level test of 5%. The coefficient of range standardization is -1.225, which passed the significance level test of 1%. The regression coefficient of relative fiscal decentralization on economic development gap is -0.663, which has passed the 1% significance level test. The coefficient of range standardization is -0.226, which has passed the 5% significance level test. This indicates that DF and DB have a mitigating effect on the imbalance and inadequacy of economic development. This means that even if the fiscal decentralization index is taken into account, our conclusion that fiscal decentralization reduces economic development imbalance is still consistent with the benchmark regression results, further proving the robustness of results.

**Table 8:** The regression results of re-measuring fiscal decentralization.

Variables	Economic imbalance	Inadequate development	Economic imbalance	Inadequate development
EI_A	EH_R	EI_A	EH_R
DF	-0.284^**^	-1.225^***^		
	(-3.264)	(-10.25)		
DB			-0.663^***^	-0.226^**^
			(-1.076)	(-9.28)
Control variables	Yes	Yes	Yes	Yes
Province fixed effect	Yes	Yes	Yes	Yes
Year fixed effect	Yes	Yes	Yes	Yes
Observed values	736	736	736	736
Adjusted R^2^	0.635	0.822	0.229	0.317

Note:^***^, ^**,*^ represents the regression coefficients at significance levels of 1%, 5%, and 10%, robust T-values are shown in parentheses.

#### 5.2.3 Instrumental variable method test

In most literature, many scholars acknowledge that the dynamics of fiscal decentralization economic development are endogenous, and we acknowledge the importance of this endogeneity issue. In previous studies, it is suggested that fiscal decentralization may not be realistic until a country reaches a certain level of economic growth. This view suggests that these countries may not be able to bear the cost of fiscal decentralization and require a strong central government to ensure economic stability. Therefore, we take measures to mitigate the possible biases that may arise from endogeneity issues. Although GMM models are commonly used to address endogeneity issues, they are typically based on stability assumptions over time. The dynamic nature of fiscal decentralization over time renders the assumption of time stability inherent in GMM models questionable. Furthermore, the influence of fiscal decentralization on economic development unfolds over an extended period, with potential long-lasting and persistent effects spanning multiple time horizons. However, the GMM model is based on a single delay period to capture endogeneity issues, which cannot accurately capture the sustained effects of fiscal decentralization on economic development. Additionally, the intricate relationship between fiscal decentralization and other explanatory variables may result in a correlation between instrumental variables and error terms, violating the exclusion restriction hypothesis. If there is endogeneity bias, the estimation results of the GMM model may have bias and inconsistency.

In analyzing economic issues, in order to effectively address potential endogeneity problems, we adopted instrumental variable estimation methods. Based on Lewbel’s theoretical framework, we selected the average fiscal decentralization of provinces other than the surveyed provinces as our instrumental variable. There is a substantial correlation between the level of fiscal decentralization within a specific province and the average fiscal decentralization in other provinces. This provides a solid foundation for the effectiveness of instrumental variables, ensuring that they can serve as effective substitutes for the original endogenous variables and capture the variability associated with the dependent variable. Secondly, it can largely avoid direct correlation with error terms in the model, thus meeting the exogeneity requirements of instrumental variables. In addition, this choice also conforms to our hypothesis of exogeneity between studying the uneven economic development within provinces and the average fiscal decentralization of external provinces. We present the regression results obtained using the instrumental variable method in columns (1) - (2) of Table 9. According to the results of the Kleiberen Paap rk LM test and the Kleiberen Paap rk Wald-F test, our selection of instrumental variables is suitable. The results showed that the regression coefficients of fiscal decentralization on EI_S and EH_S were -0.558 and -0.468, respectively, passing the significance level tests of 10% and 5%, indicating that fiscal decentralization can effectively reduce and mitigate the imbalance and inadequacy of economic development. These findings are consistent with benchmark regression results, providing important references for policy makers to promote balanced and sustainable economic development.

**Table 9:** Regression results of other robustness tests.

Variables	Instrumental variable method		Shortening sample time: 2015–2022	
EI_A	EH_R	EI_A	EH_R
AI	-0.558^*^	-0.468^**^	-0.229^***^	-0.247^*^
	(-6.38)	(-1.978)	(-6.38)	(-6.55)
Control variables	Yes	Yes	Yes	Yes
Province fixed effect	Yes	Yes	Yes	Yes
Year fixed effect	Yes	Yes	Yes	Yes
Observed values	736	736	394	394
LM test	62.275 (0.001)	52.753 (0.000)	–	–
F test	1346.278	1346.248	–	–
Adjusted R^2^	0.655	0.347	0.584	0.913

Note:***, **,* represents the regression coefficients at significance levels of 1%, 5%, and 10%, robust T-values are shown in parentheses.

#### 5.2.4 Rephrase the duration of the sample period

In addition to using instrumental variable method to address endogeneity issues, we also conducted other robustness tests. Considering the initiation of China’s fiscal revenue and expenditure categorization reform in 2015, we have narrowed the sample period to include only data from 2015 to 2022 to ensure result robustness. The regression findings are shown in columns (3) - (4) of Table 9. The regression coefficients for fiscal decentralization regarding EI_1 and EH_2 are -0.229 and -0.247, respectively, both passing the significance tests at the 1% and 10% levels. These results suggest that even within this shorter timeframe, fiscal decentralization significantly mitigates the imbalances and inadequacies in economic development. This further reinforces our research conclusions, highlighting the role of fiscal decentralization in fostering balanced economic growth. Additionally, our study confirms the effectiveness of fiscal decentralization in addressing economic disparities and deficiencies. These findings hold significant practical implications for policy formulation. However, we acknowledge the need for further research and analysis to achieve a more comprehensive understanding of fiscal decentralization's impact on economic development and to provide more targeted policy recommendations.

### 5.3 Regional heterogeneity test.

This article selects various provinces and cities across the country as representative research objects. Given the pronounced disparities in economic development across China’s various regions, fiscal decentralization plays a differentiated role, contributing to both uneven and inadequate economic growth patterns in these areas. On this basis, this project intends to analyze the impact of fiscal decentralization on the uneven and unbalanced effects of China’s economic development from the perspective of regional differences. Therefore, this project intends to divide China’s coastal areas into two major categories based on geography: north, south, and north, and conduct empirical research.

The sample is divided into two regions, north and south, for empirical testing based on the Qinling Mountains and Huaihe River. The regression analysis in Table 10 shows that, in the northern region, the coefficients related to the impact of fiscal decentralization on economic imbalance and underdevelopment are 0.284 and 0.016, respectively. However, these coefficients do not attain statistical significance. This may be related to the economic structure in the north, which is dominated by heavy industry and resource development, limiting the effectiveness of fiscal decentralization. It is necessary to adjust the industrial structure to adapt to economic transformation. In contrast, the regression coefficients of fiscal decentralization on economic disparity and underdevelopment in the southern region were -0.973 and -0.941, respectively, both of which passed the significance test. Southern residents are more open and actively participate in innovation and the digital economy. Local governments can effectively utilize their information advantages, encourage entrepreneurship, and provide public services. To ensure the effectiveness of fiscal decentralization, local governments in the south need to formulate policies, support innovation, improve the quality of public services, and promote sustainable economic development.

**Table 10:** Regression results of regional heterogeneity.

Regions	Economic imbalance	Inadequate development
EI_A	EH_R
North	0.284	0.016
	(-2.974)	(-0.539)
South	-0.973**	-0.941***
	(-5.38)	(-6.977)
Coastal	0.049	-0.225***
	(0.257)	(-3.871)
Inland	-0.497**	-0.394**
	(-6.387)	(-6.377)

Note:^***^, ^**,*^ represents the regression coefficients at significance levels of 1%, 5%, and 10%, robust T-values are shown in parentheses.

To empirically examine the effects, we segmented the sample into coastal and inland regions. The regression outcomes reveal that in coastal areas, the coefficient of fiscal decentralization on economic imbalance stands at 0.049, indicating a lack of significant mitigation in the imbalance of economic development. However, the regression coefficient on insufficient development is -0.225, which significantly indicates that economic development is suppressed. Despite the relatively advanced economy in coastal areas, there are still imbalances in industrial structure and public services. Therefore, the goal of fiscal decentralization is to narrow the regional development gap and achieve balanced and sustainable development through incentive measures. In inland areas, the regression coefficients of fiscal decentralization on economic imbalance and underdevelopment are -0.497 and -0.394, respectively, both significant. This underscores the positive role fiscal decentralization has played in alleviating economic imbalances and addressing shortcomings in inland regions. Inland regions receive transfer payments from the central government, and local governments can formulate policies to promote industrial transformation, infrastructure construction, and public service improvement. At the same time, fiscal decentralization has stimulated local governments’ innovation and competitiveness, improved governance capabilities, and promoted regional balance and sustainable development.

### 5.4 Test on the mechanism of action.

We explored the impact of fiscal decentralization on the balance of economic development and its underlying mechanisms. Theoretical discussions indicate that fiscal decentralization may help alleviate economic imbalances and deficiencies by enhancing financial support for technological innovation and improving the administrative framework. To validate this hypothesis, this article identified technological support and the administrative environment as key variables for empirical analysis. The regression results shown in Table 11 reveal that in column (1), fiscal decentralization exhibits a statistically significant (at 5% level) positive coefficient of 0.094 on technological support, thereby confirming its favorable influence on technological enhancement. From this, it can be seen that fiscal decentralization can enhance local governments’ support for technological progress, thereby promoting economic innovation and development. However, at the same time, we note that in columns (1) and (2), although the coefficient of technological support in alleviating economic imbalances is -0.229 and has passed the 5% significance level test, this suggests that technological support is an important way for fiscal decentralization to affect economic development balance. Additionally, the regression outcomes in columns (1) and (3) reveal that when considering technological support as a mediator, fiscal decentralization exerts a notable negative influence on economic underdevelopment. This underscores the pivotal role of technological support as a conduit between fiscal decentralization and economic growth. Furthermore, column (4) of the regression analysis demonstrates that fiscal decentralization boasts a statistically significant (at 1% level) positive coefficient of 0.633 on the administrative environment, indicating its effectiveness in enhancing administrative efficiency and optimizing the quality of government administration. The regression analysis in columns (4) and (5) indicates that the coefficient of the administrative environment on economic imbalance is -0.228, which is statistically significant at the 10% level. This supports the idea that the administrative environment acts as a mediating factor in the relationship between fiscal decentralization and the balance of economic development. Likewise, in columns (4) and (6), the regression coefficient of the administrative environment on economic underdevelopment is -0.294, also significant at the 10% level, indicating that the administrative environment plays a crucial intermediary role in how fiscal decentralization influences economic development. Based on the above results, our hypothesis H1 has been validated. Therefore, it can be concluded that strengthening technological support and optimizing the administrative environment are important links in implementing fiscal decentralization policies. By enhancing local government funding for technological development and improving the administrative environment, fiscal decentralization promotes economic innovation and growth, thereby promoting the balance of economic development.

**Table 11:** Regression results of mechanism of action.

Variables	Tech support	Economic imbalance	Inadequate development	Administrative environment	Economic imbalance	Inadequate development
TS	EI_A	EH_R	CF	EI_A	EH_R
AI	0.094^**^	-0.229^**^	-0.394^**^	0.633^***^	-0.228^*^	-0.294^*^
	(6.387)	(-3.584)	(-6.387)	(5.28)	(-3.684)	(-3.287)
TS		-0.638	-0.838^*^			
		(-0.294)	(-5.28)			
CF					-0.284^**^	-0.467^***^
					(-6.355)	(1.284)
Control variables	Yes	Yes	Yes	Yes	Yes	Yes
Province fixed effect	Yes	Yes	Yes	Yes	Yes	Yes
Year fixed effect	Yes	Yes	Yes	Yes	Yes	Yes
Observed values	736	736	736	736	736	736
Adjusted R^2^	0.895	0.361	0.294	0.883	0.526	0.954

Note:^***^, ^**,*^ represents the regression coefficients at significance levels of 1%, 5%, and 10%, robust T-values are shown in parentheses.

### 5.5 Further analysis.

#### 5.5.1 Reassessment of the adjustment effect

Having conducted a comprehensive examination of fiscal decentralization’s influence on economic development and the underlying dynamics at play, we delved deeper into the interactive effects between fiscal decentralization and other variables, meticulously considering potential confounding factors that may influence our findings. To avoid the problem of multicollinearity, we adopted centralized processing of interaction variables. Using this approach, we incorporated interaction terms between fiscal decentralization and industrial structure, as well as between fiscal decentralization and fiscal pressure, to examine their effects on uneven and inadequate economic development.

The regression results in Table 12 illustrate the intricate interactions among fiscal decentralization, industrial structure, and fiscal pressure, along with their effects on economic development. In addressing economic imbalances, the interaction between fiscal decentralization and industrial structure demonstrates a significant impact (coefficient -2.571, p < 0.01), suggesting that optimizing the industrial structure notably strengthens the capacity of fiscal decentralization to mitigate economic imbalances. Similarly, for the insufficient economic development, this interaction also showed a positive impact (coefficient -0.553, p < 0.05), emphasizing that optimizing industrial structure effectively alleviates the problem of insufficient development by improving resource allocation efficiency and promoting regional balance. On the other hand, the interaction analysis between fiscal decentralization and fiscal pressure shows that the combined effect of the two has a significant effect on alleviating economic imbalances (coefficient -0.538, p < 0.1) and underdevelopment (coefficient -2.756, p < 0.01). This reveals that fiscal pressure, as an intrinsic driving force, can regulate and strengthen the positive role of fiscal decentralization in addressing economic development challenges. These findings provide valuable references for policy makers, aiming to promote more balanced and comprehensive economic development through comprehensive policies.

**Table 12:** Regression results of adjustment effect.

Variables	Economic imbalance	Inadequate development	Economic imbalance	Inadequate development
EI_A	EH_R	EI_A	EH_R
AI	0.394***	-0.459***	-0.394*	-0.113*
	(3.975)	(3.387)	(-3.587)	(-3.294)
IB	-0.297***	-0.635***		
	(-5.994)	(-2.375)		
AI × IB	-2.571***	-0.553**		
	(-6.387)	(-6.294)		
PS			0.539*	0.733***
			(2.257)	(8.58)
AI × PS			-0.538*	-2.756***
			(0.977)	(-8.29)
Control variables	Yes	Yes	Yes	Yes
Province fixed effect	Yes	Yes	Yes	Yes
Year fixed effect	Yes	Yes	Yes	Yes
Observed values	736	736	736	736
Adjusted R2	0.639	0.918	0.581	0.883

Note:^***^, ^**,*^ represents the regression coefficients at significance levels of 1%, 5%, and 10%, robust T-values are shown in parentheses.

#### 5.5.2 Examination by Panel threshold effect

In this study, we focus on the deep-seated effects of fiscal decentralization on economic disparities and resource shortages, and further explore the effects of tax competition as a potential moderating factor. We employed a dual threshold model to discern how fiscal decentralization influences economic imbalances and inadequacies under varying tax competition intensities. Following equation (5), empirical analyses were performed using a dual threshold regression approach to assess the impacts of fiscal decentralization across different tax competition spectra. The tax competition measure was utilized as a threshold variable, splitting the data into three segments to gauge the moderating influence of tax competition on fiscal decentralization outcomes.

Table 13 presents the findings from the panel threshold regression analysis, revealing notable variations in the influence of fiscal decentralization on economic development imbalances and insufficiencies across distinct tax competition intervals. In interval 1 of tax competition, the influence of fiscal decentralization on economic disparities and shortfalls is minimal and lacks statistical significance, with coefficients at 0.025 and 0.035, respectively; however, these do not reach sufficient levels of significance. As tax competition moves into interval 2, the suppressive effect of fiscal decentralization on economic development imbalances and insufficiencies begins to surface, evidenced by regression coefficients of -0.127 and -0.225, which pass the significance tests at the 10% and 5% levels, respectively. This suggests that fiscal decentralization can effectively mitigate economic development imbalances within this range. In the third interval of tax competition, fiscal decentralization exhibits an even more pronounced suppressive effect on economic imbalances and insufficiencies, with coefficients of -0.365 and -0.443, both statistically significant at the 5% level. This indicates that with the intensification of tax competition, the positive effect of fiscal decentralization is further enhanced, and the promoting effect on economic development is more pronounced. The degree of tax competition is an important factor affecting the unbalanced and insufficient inhibitory effect of fiscal decentralization on economic development. The intensification of tax competition can promote effective competition among local governments by optimizing resource allocation and enhancing the role of fiscal decentralization in promoting balanced economic development.

**Table 13:** Panel threshold regression results.

Interval	Economic imbalance	Inadequate development
EI_A	EH_R
Interval 1 (CB<ω_*1*_)	0.025	0.035
	(0.43)	(0.294)
Interval 2 (ω_*1*_* ≤ CB<*ω_*2*_)	-0.127^*^	-0.225^**^
	(-2.375)	(-3.594)
Interval 3 (CB>ω_*2*_)	-0.365^**^	-0.443^**^
	(-6.287)	(-5.294)
Control variables	Yes	Yes
Province fixed effect	Yes	Yes
Year fixed effect	Yes	Yes
Observed values	736	736

Note:***, **,* represents the regression coefficients at significance levels of 1%, 5%, and 10%, robust T-values are shown in parentheses.

Table in S1 Table (**Comparison with other articles**) presents a comparison between five different research articles, all of which focus on the impact of fiscal decentralization on economic growth. The table compares the research variables, research methods used, and main conclusions drawn from these articles from multiple dimensions. For example, the article ‘The Impact of Fiscal Decentralization on Economic Growth: A Comparative Analysis of Selected African and OECD Countries’ used two-stage least squares (2SLS), generalized moment estimation (GMM), limited information maximum likelihood (LIML), and ordinary least squares (OLS) methods, and found that the decentralization of fiscal power has a significant positive impact on economic growth. Our study demonstrates that fiscal decentralization effectively addresses the imbalances and deficiencies in economic development, thereby advancing common prosperity. By examining the variations in variable selection, methodological approaches, and key findings across different studies, a multidimensional perspective is offered for understanding the relationship between fiscal decentralization and economic growth. Collectively, these studies highlight the intricate effects of fiscal decentralization on economic growth, along with the differences in these effects across various countries, regions, and economic sectors.

The comparison of research data with other articles is shown in Table 14.

**Table 14:** Comparative analysis of research data.

Title	Data sources	Data Sample	Data Time Range	Data result analysis
Decentralization and Political Institutions	IMF’s Government Finance Statistics; Database on Political Institutions; World Development Indicators by the World Bank	Data from 95 countries	1975-2000	In developing countries, for every 10% increase in decentralization, if the age of the political party is less than half the standard deviation of the average age, it will lead to a decline of more than 30% in economic growth over 25 years.
Fiscal Decentralization and Economic Growth: Spending Versus Revenue Decentralization	OECD General Government Accounts; IMF (2001) Government Finance Statistics (GFS); Eurostat data	23 countries of the Organization for Economic Cooperation and Development (OECD).	1972-2005	Direct spending decentralization negatively correlates with economic growth (coef. -0.050). Govt revenue/GDP ratio also has a negative impact (-0.052), implying fiscal burden hurts growth. In OECD nations, cutting spending decentralization & boosting local revenue decentralization may boost economic growth.
Effect of Fiscal Decentralization Policy of Regional Economic Imbalances towards Economy Growth in Eastern Indonesia	General Allocation Fund (GAF)	16 provinces in East Indonesia	2001-2010	The article points out that between 2009 and 2018, the fiscal decentralization funds allocated through the Balanced Fund (BF) increased by 20.86%, while per capita GDP income also increased by 20.41% annually. The relationship between fiscal decentralization and regional imbalance is not consistent. For example, from 2009 to 2011, when fiscal decentralization funds decreased, the imbalance tended to increase; In 2012–2013, although fiscal decentralization funds increased again, the imbalance also tended to increase.
Rising Chinese regional income inequality: The role of fiscal decentralization	Chinese Statistics Yearbook (2005–2008); Comprehensive Statistical Data and Materials on 50 Years of New China; Statistical yearbooks of each province	Data from 28 provinces in China from 1978 to 2007. Hainan, Chongqing, and Qinghai were excluded due to a lack of income data and financial information during the period of 1978–1990.	1978-2007	The article states that a 10% rise in local govt expenditure share hikes regional income inequality by avg. 3.54%, whereas pre-1994 tax reform, a 10% autonomy boost reduced it by 2.48%. Post-1994 reform, a 10% autonomy increase led to a 0.15% rise in inequality. The CovAP coefficient (-0.0915) suggests more even fiscal independence distribution among provinces curbs regional income inequality.

## 6 Conclusion and future research directions

### 6.1 Conclusion.

Previous studies have shown that fiscal allocation rights play a crucial role in economic management and social governance, enabling regions to develop, utilize, and allocate resources based on their respective resources and income potential, thereby achieving maximum economic benefits and social benefits. Fiscal decentralization has a positive effect on narrowing regional economic disparities and promoting economic development, thereby promoting regional economic development and improving and social benefits. This study examines provincial panel data from China covering the years 2013–2022. Through regression analysis, robustness checks, regional heterogeneity assessments, and mechanism evaluations, it investigates the effects of fiscal decentralization on uneven and inadequate economic development, as well as potential pathways to achieve common prosperity and the level of social governance.

The research results show that fiscal decentralization has played a key role in alleviating the economic growth gap between inter provincial cities and promoting balanced regional economic development. This policy mechanism not only promotes the formation of a more balanced and inclusive economic pattern, but also effectively promotes the achievement of the goal of common prosperity. Through regional difference analysis, we found that fiscal decentralization has a more significant positive impact on the southern and inland regions, highlighting its role in promoting economic equality in different geographical areas. Therefore, adopting differentiated fiscal policies and measures based on the characteristics and needs of different regions is an important way to achieve balanced economic development and common prosperity and social governance.

In addition, mechanism testing shows that strengthening technical support and optimizing the administrative environment are effective means of fiscal decentralization to alleviate economic imbalances and deficiencies. Improving the administrative environment helps to enhance government governance efficiency, while increasing investment in technology can improve industrial competitiveness and innovation capabilities, thereby promoting comprehensive economic development. Meanwhile, the efficacy evaluation conducted through adjustment testing shows that restructuring the industrial sector and alleviating fiscal pressure are key strategies for mitigating the adverse effects of fiscal decentralization. By optimizing the industrial structure to achieve economic upgrading and transformation, as well as managing financial pressure to avoid resource waste and abuse, we can further improve the quality and efficiency of economic development. It is worth noting that the threshold effect test also found that greater tax competition not only intensifies the inhibitory effect of fiscal decentralization on imbalanced and insufficient economic development, but also improves the efficiency of market resource allocation and to some extent narrows the gap. These findings provide a new perspective for us to understand the complex role of fiscal decentralization in economic development, and provide useful references for future policy formulation and implementation.

However, while fiscal decentralization promotes economic development, it may also exacerbate regional inequality and funding shortages. Under fiscal decentralization, local governments may compete for limited resources and funds for their own interests, especially when the overall government budget is limited. This competition may exacerbate regional inequality and uneven resource allocation. Therefore, future policy formulation should pay more attention to the fair distribution of resources, avoiding certain regions from obtaining excessive resources due to political considerations while other regions face the problem of insufficient funding. By optimizing fiscal decentralization policies and ensuring the rational allocation of resources, it can further promote the balanced development of regional economy and common prosperity and social governance.

### 6.2 Future research directions.

Future research can further explore the long-term effects of fiscal decentralization in different economic contexts, especially in response to global economic changes and domestic economic structural adjustments. We can also pay attention to the interaction between fiscal decentralization and social policies, and explore how to further enhance the effectiveness of fiscal decentralization through the optimization of social security and public services. Considering the importance of technological progress in economic development, we can also explore how to support technological innovation through fiscal policies to achieve more sustainable and inclusive economic growth.

In addition, future research should also focus on the fairness of fiscal decentralization in resource allocation. How to ensure the rational allocation of resources under fiscal decentralization, and avoid certain regions obtaining excessive resources due to political considerations while other regions face the problem of insufficient funds, is a topic worthy of in-depth exploration. By further studying the relationship between fiscal decentralization and resource allocation, more comprehensive references can be provided for policy makers to promote balanced regional economic development and common prosperity.

## Supporting information

S1 Table Comparison with other articles. (DOCX)

## References

[1] Arabatzis G, Polyzos S. Contribution of natural and socio-cultural resources in tourism development of Mainland Greek prefectures: A typology. J Environ Prot Ecol. 2008;9(2):446-–464.

[2] Sidik M. Balancing of Finance Central and Region for Fiscal Decentralization. Theory and Applied. Seminar paper in Gajahmada University; 2002. Yogyakarta: Local Politic Programme Study Postgraduate Gajahmada University.

[3] Soejoto A, Subroto WT, Suyanto Y. Fiscal decentralization policy in promoting Indonesia human development. International Journal of Economics and Financial Issues. 2015;5(3):763-–771.

[4] Ntanos S, Skordoulis M, Kyriakopoulos G, Arabatzis G, Chalikias M, Galatsidas S, et al. Renewable energy and economic growth: Evidence from European countries. Sustainability. 2018;10(8):2626. doi: 10.3390/su10082626

[5] Pommerehne WW. Quantitative aspects of federalism: A study of six countries. Diskussionsbeiträge. 1976.

[6] Panizza U. On the determinants of fiscal centralization: Theory and evidence. Journal of Public Economics. 1999;74(1):97–139. doi: 10.1016/s0047-2727(99)00020-1

[7] Qian Y, Weingast BR. China’s transition to markets: market-preserving federalism, chinese style. The Journal of Policy Reform. 1996;1(2):149–85. doi: 10.1080/13841289608523361

[8] Akai N, Sakata M. Fiscal decentralization contributes to economic growth: evidence from state-level cross-section data for the United States. Journal of Urban Economics. 2002;52(1):93–108. doi: 10.1016/s0094-1190(02)00018-9

[9] Lamba A, Allo PK, Lamba RA. Effect of fiscal decentralization policy of regional economic imbalances towards economy growth in Eastern Indonesia. IJSSH. 2019;3(2):112–27. doi: 10.29332/ijssh.v3n2.298

[10] Hung NT, Thanh SD. Fiscal decentralization, economic growth, and human development: Empirical evidence. Cogent Economics & Finance. 2022;10(1). doi: 10.1080/23322039.2022.2109279

[11] Jin H, Qian Y, Weingast BR. Regional decentralization and fiscal incentives: Federalism, Chinese style. Journal of Public Economics. 2005;89(9–10):1719–42. doi: 10.1016/j.jpubeco.2004.11.008

[12] Song Y. Rising Chinese regional income inequality: The role of fiscal decentralization. China Economic Review. 2013;27:294–309. doi: 10.1016/j.chieco.2013.02.001

[13] Wang Q, Nayak BS. Two decades of fiscal decentralization and regional economic growth in China. In: China: The great transition: from agrarian economy to technological powerhouse. Singapore: Springer Nature Singapore; 2023;9–37. doi: 10.1007/978-981-99-0051-0_2

[14] Liu Y, Martinez-Vazquez J, Wu AM. Fiscal decentralization, equalization, and intra-provincial inequality in China. Int Tax Public Finance. 2016;24(2):248–81. doi: 10.1007/s10797-016-9416-1

[15] Gemmell N, Kneller R, Sanz I. Fiscal decentralization and economic growth: Spending versus revenue decentralization. Economic Inquiry. 2013;51(4):1915–31. doi: 10.1111/j.1465-7295.2012.00508.x

[16] Bird RM, Vaillancourt F. Fiscal decentralization in developing countries: an overview. Fiscal Decentralization in Developing Countries. 1998;1:1-–48.

[17] Tan E, Avshalom-Uster A. How does asymmetric decentralization affect local fiscal performance?. Regional Studies. 2021;55(6):1071–83. doi: 10.1080/00343404.2020.1861241

[18] Oates WE. Fiscal decentralization and economic development. National Tax Journal. 1993;46(2):237–43. doi: 10.1086/ntj41789013

[19] Neyapti B. Fiscal decentralization and deficits: International evidence. European Journal of Political Economy. 2010;26(2):155–66. doi: 10.1016/j.ejpoleco.2010.01.001

[20] Hao Y, Liu J, Lu Z-N, Shi R, Wu H. Impact of income inequality and fiscal decentralization on public health: Evidence from China. Economic Modelling. 2021;94:934–44. doi: 10.1016/j.econmod.2020.02.034

[21] Dvinin D, Davankov A, Plaksina A. Methodological toolkit for assessing the impact of renewable energy on the balanced development and preservation of the regional carbon cycle. IOP Conf Ser: Earth Environ Sci. 2022;1070(1):012010. doi: 10.1088/1755-1315/1070/1/012010

[22] Mavraki C, Arabatzis G, Kantartzis A, Malesios C. Fostering regional development in eastern Macedonia and Thrace, Greece, through road transport projects. Economic Analysis and Policy. 2020;65:56–67. doi: 10.1016/j.eap.2019.11.008

[23] Buser W. The impact of fiscal decentralization on economics performance in high-income OECD nations: an institutional approach. Public Choice. 2011;149(1–2):31–48. doi: 10.1007/s11127-011-9827-6

[24] Slavinskaitė N. Fiscal decentralization and economic growth in selected european countries. Journal of Business Economics and Management. 2017;18(4):745–57. doi: 10.3846/16111699.2017.1292312

[25] Feng G, Shulian X, Renjin S. Does fiscal decentralization promote or inhibit the improvement of carbon productivity? Empirical analysis based on China’s data. Front Environ Sci. 2022;10. doi: 10.3389/fenvs.2022.903434

[26] Safi A, Wang Q-S, Wahab S. Revisiting the nexus between fiscal decentralization and environment: evidence from fiscally decentralized economies. Environ Sci Pollut Res Int. 2022;29(38):58053–64. doi: 10.1007/s11356-022-19860-1 35364787

[27] Polyzos S, Arabatzis G. Determinant factors of tourist attractiveness of Greek prefectures. International Journal of Sustainable Development and Planning. 2008;3(4):343-–366. doi: 10.2495/SDP-V3-N4-343-366

[28] Liu Y, Martinez-Vazquez J, Wu AM. Fiscal Decentralization, Equalization, and Intra-Provincial Inequality in China. International Center for Public Policy Working Paper. 2014,14-–32. https://scholarworks.gsu.edu/icepp/210

[29] Cavusoglu T, Dincer O. Does decentralization reduce income inequality? Only in rich states. Southern Economic Journal. 2015;82(1):285–306. doi: 10.1002/soej.12047

[30] Scott Z. Decentralisation, local development and social cohesion: an analytical review. GSDRC Research Paper. 2009;5:1-–22.

[31] Labutková Š, Bednářová P, Hovorková Valentová V. Economic inequalities and the level of decentralization in European Countries: Cluster analysis. CER. 2016;19(4):27–46. doi: 10.1515/cer-2016-0028

[32] Siburian ME. Fiscal decentralization and regional income inequality: Evidence from Indonesia. Applied Economics Letters. 2019;27(17):1383–6. doi: 10.1080/13504851.2019.1683139

[33] Hanif I, Wallace S, Gago-de-Santos P. Economic growth by means of fiscal decentralization: An empirical study for federal developing countries. Sage Open. 2020;10(4). doi: 10.1177/2158244020968088

[34] Jin Y, Rider M. Does fiscal decentralization promote economic growth? An empirical approach to the study of China and India. JPBAFM. 2020;34(6):146–67. doi: 10.1108/jpbafm-11-2019-0174

[35] Lin JY, Liu Z. Fiscal decentralization and economic growth in China. Economic Development and Cultural Change. 2000;49(1):1–21. doi: 10.1086/452488

[36] Digdowiseiso K, Murshed SM, Bergh SI. How effective is fiscal decentralization for inequality reduction in developing countries?. Sustainability. 2022;14(1):505. doi: 10.3390/su14010505

[37] Nguyen HT, Vo THN, Le DDM, Nguyen VT. Fiscal decentralization, corruption, and income inequality: Evidence from Vietnam. The Journal of Asian Finance, Economics and Business. 2020;7(11):529–40. doi: 10.13106/JAFEB.2020.VOL7.NO11.529

[38] Weingast BR. Second generation fiscal federalism: The implications of fiscal incentives. Journal of Urban Economics. 2009;65(3):279–93. doi: 10.1016/j.jue.2008.12.005

[39] Zhou C, Zheng H, Wan S. Industrial structure, employment structure and economic growth — Evidence from China. Sustainability. 2023;15(4):2890. doi: 10.3390/su15042890

[40] Chang Y, Wu P. Influence of fiscal decentralization, fintech, and mineral resources on green productivity of G5 countries. Resources Policy. 2024;89:104509. doi: 10.1016/j.resourpol.2023.104509

[41] Sima M, Liang P, Qingjie Z. The impact of fiscal decentralization on economic growth: A comparative analysis of selected African and OECD countries. Heliyon. 2023;9(9):e19520. doi: 10.1016/j.heliyon.2023.e19520 37809777 PMC10558754

[42] Faguet J-P. Decentralization and governance. World Development. 2014;53:2–13. doi: 10.1016/j.worlddev.2013.01.002

[43] Lewbel A. Constructing instruments for regressions with measurement error when no additional data are available, with an application to patents and R&D. Econometrica. 1997;65(5):1201. doi: 10.2307/2171884

