# Supplementary material for: Chinese economic development difference factors empirical study
Source: PLoS One. 2025 May 23;20(5):e0319957. doi: 10.1371/journal.pone.0319957 (PMC12101844; doi:10.1371/journal.pone.0319957)
Supplement: S1 Table — (DOCX) [file pone.0319957.s001.docx]

**S1 Table. Comparison with other articles.**

| Article | Variable | Method | Conclusion |
| --- | --- | --- | --- |
| The impact of fiscal decentralization on economic growth: A  comparative analysis of selected African and OECD countries[1] | Expenditure decentralization, Ethnic Fractionalization, Government Expenditure % of GDP, Inflation, Average Tax Rate, etc. | two-stage least squares,  Generalized Method of Moments, Limited Information Maximum Likelihood, Ordinary Least Squares. | The decentralization of fiscal power has had a significant positive impact on economic growth. |
| Does fiscal decentralization  promote economic growth?  An empirical approach to the study  of China and India[2] | Fiscal decentralization, Growth rate in real GRP per capita, Index of horizontal fiscal equalization, etc. | Two step GMM simultaneous equation model, Growth equation, Equalization equation. | Decentralization of expenditure has a positive and statistically significant impact on fiscal equalization in both countries. |
| Tax reform, fiscal decentralization, and regional economic growth: New  evidence from China[3] | Primary sector GDP growth rate,  Secondary sector GDP growth rate,  Tertiary sector GDP growth rate  Revenue decentralization,  Expenditure decentralization, etc. | Exponential panel data model, Euantitative regression method, Fixed effect model. | There are differences in the impact of fiscal decentralization on the economic growth of various provinces in China. Among them, fiscal decentralization has the greatest impact on the secondary industry, and in the secondary industry, the impact of fiscal decentralization shows a U-shaped relationship. In addition, the impact of fiscal decentralization on economic growth also depends on the economic growth rate of each province. |
| Fiscal Decentralization and Regional Economic Growth[4] | Economic growth, Fiscal decentralization, Private investment, and Labor force. | Multiple regression analysis using the method Fixed Effect Model (FEM) | When implementing fiscal decentralization, it is necessary to ensure that local governments can effectively carry out their allocation functions to promote economic growth. Fiscal decentralization has a positive effect on promoting local economic growth. |
| Empirical Study of the Fiscal Policy Impact on Economic Growth[5] | Population growth, GDP per capita growth rate,  Fiscal decentralization indicato, Human development index (HDI),  Investment as % of GDP ratio,  Population growth rate ratio, etc. | Panel Data Analysis,  Econometric Modeling,  Descriptive Statistics,  Regression Analysis,  Robust Checks, etc. | Countries with higher levels of fiscal decentralization typically have stronger economic growth potential; Countries with lower levels of fiscal decentralization often face greater development obstacles. |

References

1. Sima M, Liang P, Qingjie Z. The impact of fiscal decentralization on economic growth: A comparative analysis of selected African and OECD countries. Heliyon. 2023;9(9). <https://doi.org/10.1016/j.heliyon.2023.e19520>
2. Jin Y, Rider M. Does fiscal decentralization promote economic growth? An empirical approach to the study of China and India. J Public Budgeting, Accounting & Financ Manage. 2022;34(6):146-167. <https://doi.org/10.1108/JPBAFM-11-2019-0174>
3. Yang Z. Tax reform, fiscal decentralization, and regional economic growth: New evidence from China. Econ Modelling. 2016;59:520-528. https://doi.org/10.1016/j.econmod.2016.07.020
4. Sasana H. Fiscal decentralization and regional economic growth. Econ Develop Anal J. 2019;8(1):108-119. <https://doi.org/10.15294/edaj.v8i1.29879>
5. Pasichnyi M, Kaneva T, Ruban M, et al. The impact of fiscal decentralization on economic development. Invest Manage Financ Innovations. 2019;16(3). https://doi.org/10.2139/ssrn.3099970
